# An automated guide to COVID-19 and future pandemic prevention and management

**DOI:** 10.1186/s43067-023-00084-3

**Published:** 2023-03-16

**Authors:** George Emeka Okereke, Okechukwu Azegba, Emmanuel Chukwudi Ukekwe, Stephenson Chukwukanedu Echezona, Agozie Eneh

**Affiliations:** 1 Department of Computer Science, University of Nigeria, Nsukka, Enugu State, South East Nigeria; 2 Alex Ekwueme Federal University Ndufu-Alike, P.M.B. 1010, Abakiliki, Ebonyi State Nigeria

**Keywords:** COVID-19, CoFighter, Pandemic, Mobile application, Health crisis, COVID-19 management

## Abstract

In this paper, we present CoFighter, a mobile application for prevention and management of COVID-19 and other related pandemics in the globalized world. We took advantage of the proliferation of mobile smart devices in every home to design and implement an Android application for COVID-19 and similar pandemics. Since the outbreak of COVID-19 pandemic in 2019, there has been even more serious pressures on governments and health institutions on the best way to provide appropriate and reliable guide to individuals on how to contain the virus and similar pandemics in the future. Citizens have not been adequately informed of the various provisions and guides by their governments and the wide usage of social media had led to the spread of fake news, misinformation and conspiracy theories. It therefore becomes very necessary to develop a dynamic information repository in the form of a mobile application to help combat the spread of any pandemic whenever the need arises. The application provides information on COVID-19, vaccine challenges, prevention guides and cases management and timely updates to keep citizens properly and adequately informed. It makes provision for future similar pandemics that could throw the world into chaos as the CORONA virus did in 2019. The weaknesses and challenges observed in most popularly existing COVID-19 applications were highlighted and implemented in CoFighter. CoFighter provides users, governments and health workers with a platform not only to manage COVID-19 and other similar pandemics in the future, but also helps frontline health workers to better manage the pandemics. The developed application runs on an Android device with Android version 4.2 or higher and can be used not only to manage COVID-19 pandemic, but also to manage economic crisis and similar future pandemics. CoFighter is available via the Repository: https://github.com/OkeyIsOkay/CoFighter-Project.

## Introduction

The outbreak of COVID-19 pandemic—an infectious disease which originated in China in December 2019—caused by SARS-CoV-2 (an infectious respiratory syndrome coronavirus 2), led to health crisis worldwide [1, 2]. It was on January 11, 2020, that the first known death from the virus was reported from China [3, 4]. This led to an urgent search for solutions to contain the violent spread of the pandemic as the number of deaths increased geometrically [5, 6]. Most of the approaches have been on developing contact tracing applications to identify infected persons or those at risk of getting infected. While this approach has helped in identifying and isolating suspected cases, it has not solved the impact of information shortage on the COVID-19. Despite several guidelines issued by [7, 8], people still need to come out during the lockdown to search for food and so there is need for adequate information on the security strategies to enable them play safe especially in Africa where most governments are not prepared for emergencies. In some countries and states boarders with lockdown or curfew, stranded travellers are seen locked out or forced to quarantine [9, 10, 8]. There are massive snub of the vaccination exercise in most countries because of no proper information. Large parts of the populace have no access to a dedicated information source on COVID-19 and depended on social media which are not fully trusted and may not be dynamic most of the times [11]. The application called CoFighter is a mobile application for COVID-19 and its variants prevention. It arms users with comprehensive information and recourses on COVID-19.The dashboard contains automatic updates on COVID-19 cases for the selected country. The application user can check any country, state, city or municipal COVID-19 status through the status guide. The system allows users to register for COVID-19 vaccination and also check vaccination status of an individual. It manages COVID-19 cases from the various COVID-19 centres through automatic updates. The application helps to curb the proliferation and penetration of fake news from various social media as users can confirm any breaking news using the application. This mobile application provides timely response and dynamic information which helps prevent panic and lack of trust in government. It engages users during the time of pandemic to know what their government is doing so as to prevent agitations and demonstrations. The application helps create adequate awareness on adherence to the COVID-19 and similar pandemic rules and prevention guides.

The introduction is presented in section "Introduction"; section "Related works" is a detailed survey of related works; analysis of the proposed system is in section "Analysis of the proposed system"; section "Comparative analysis of CoFighter with other related applications" did a comparative analysis of CoFighter with seven related applications; conclusion and recommendations are presented in sections "Results" and "Conclusion" respectively.

## Related works

A web application called COVID-19 WATCHER was presented in [12]. It is a web-based application developed to update the USA residents on COVID-19 outbreak in their area. The methods include accessing daily updated public dataset in the country: The New York Times COVID-19 data, Johns Hopkins University COVID-19 data and COVID Tracking Project data. The application was developed using R. Shiny, while data visualization is achieved through Ggplot2 package. Data updates from the sources are checked every hour and is downloaded to the server which was linked with the web resources. The online resource displays data on COVID-19 from all the metropolitans and counties in the USA. It ranks the affected areas using plots by considering testing capacity, cases and deaths.

An *Android* application was developed by [13, 14] to create awareness and assist the people during the time of COVID-19 pandemic. Their system contains three sections, the Volunteer, the User and the Authority sections. Authors developed *Android* application using Android studio as IDE. Firebase was the database used to store and sync user data in real time. The main application was built using Java. The Volunteer section allows individual or organization to make donations. The User section allows users to check for symptoms, check prevention information and access live case updates. Authority section gathers information about the users and uses it to make helpful decisions. The application was developed with Java while Flutter, PHP and JavaScript were used in our implementation. It also provided prevention awareness and live case update to its users.

The authors in [15] proposed contact tracing using smartphone-based technology. The smartphone-based contact tracing application uses Global Navigation Satellite System (GNSS) receiver for tracing when in an open environment and bluetooth technology when the user is indoors. The technology alerts every registered user who might have been in contact with a confirmed infected user. To do this, the system needs to retrieve the MAC address of another close user before performing matching process. On its policy making called lock down proposal model, it analyses the geographical information of the infected user. The web portal for the system administrator was designed and implemented using PHP, JAVAScript, HTML5 and API from Google map. K-means algorithm is an unsupervised machine learning algorithm used in predicting the lockdown decision of an area. This helped the authority in tracing contacts while informing users when in danger of getting infected. Policy making was made easy through the lockdown prediction platform. Both research works took advantage of the availability of smartphones in virtually every home to tackle the pandemic and involved users registering for effective delivery.

A dataset that provides updates and tracks government response and policy on the COVID-19 outbreak was presented in [16]. It gets information on government and the measures taken and when. This helps policy makers and residents or users understand government responses, leading to efforts to fight the pandemic. Oxford Covid-19 Government Response Team (OxCGRT) collects information from the public on more than 19 indicators. This data is collected by a team of over 190 volunteers from the Oxford University and is updated regularly. Data are collected through Azure-based SQL on a customized website and analysed using Stata. It tracks more than 19 indicators which are used to determine the government policies around the world. Some of the indicators are school closure, international travel controls, public event cancellation, and public transport closure among others. Over 100 countries were included in the study. The main idea is to assist government policy makers and researchers know the effect of different government interventions and to find out the triggers for placing more or less strict measures during the COVID-19.

A cross platform application (mobile and web) which creates awareness on COVID-19 to a reasonable extent was very helpful [17]. The system was divided into two: the front end and the backend components. The front end is user interface that makes request to the backend, a server hosted in the cloud. The server side contains a database that is populated with data from the public API. Chart.js was used to create pictorial representations of cases. The system was designed using Flutter framework. The application which was developed in India provides users with COVID-19 case status like, active case and death case in the country, state and city levels. It uses a pictorial representation (bar graph) to summarize updates on a selected state or city. With the system, the health status of a city or state can be predicted. A model that allows daily report on individual’s symptoms of COVID-19, test outcomes, and geographical information of the users was proposed by [18]. From the analysis of the symptoms reported, it was strongly believed that having symptoms like persistence cough, anosmia, weakness, and loss of appetite together may be able to identify person with COVID-19. Again symptoms like loss of smell and taste is a good predictor of COVID-19 together with other known symptoms such as high body temperature and cough that persists. pRoc and epiR are the packages of R used for the predicting model. The research focused on predicting positive or negative COVID-19 test through the track and analysis of symptoms. This helps on advising individuals with mild symptoms to self- isolate and also helps in reducing cost in using test centres.

Singapore was one the first to come up with an official mobile-based application for COVID-19 contact tracing called TraceTogether [19]. The application alerts the user after being exposed to the virus through contacts or close contacts with other users of the application. It uses Bluetooth technology to locate other users within a particular range. The application can only be used in Singapore and is aimed at fighting the spread of the virus and ease of tracing contacts. COVIDSAFE, an Australian contact and trace application was modelled after TraceTogether. It uses Bluetooth technology and can log users’ contact which can be used to inform other users who may have come in contact with an infected person. These applications were able to add another important dimension in fighting the pandemic other than COVID-19 case updates tracking system. While these applications are argued to be very good at what it does, the security of sensitive data like address and location of users remain a big concern. It was argued that such an application will be less important when everyone is isolating. Users of TraceTogether have also reported serious smart phone battery drain as the application has to be launched (remains open on the screen) before it could function. The authors in [20] studied the various technologies that can be used in fighting COVID-19 pandemic. Artificial intelligence, social media platforms, virtual reality, block chain, 5G cellular technology and smart application, etc., were among the technologies studied. They identified the strength and weakness of each of the technologies. On 5G cellular technology and smart application, advantages include remote consultation, smart thermometers, etc. The challenges are lack of health privacy, huge capital requirements among others. The authors argued that there is still need for further improvement on these technologies for tracking, monitoring and creating awareness on COVID-19.

The acceptability level of the various mobile-based application used in contact tracing was surveyed by authors in [21]. The aim was to examine the user acceptability level of the applications in five selected countries. A multi-country study to quantify user support for the contact tracing application was used in the selected countries. An online survey was conducted in France, Italy, United Kingdom, Germany and the USA. The study was about intention of users to install the application which is voluntary installation vs. default installation by smartphone providers and how it differs across users and countries. Strong acceptability for the application installed under both conditions (voluntary installation and automatic installation) in all the countries and across all the categories of users of the population was discovered. Factors that may hinder the adoption of the application were also investigated and concerns about security and privacy coupled with lack of trust in government were found. It was observed that there was high willingness from the users to install the application. Again, from the available result, contact tracing application may be a good approach to contain the COVID-19.

Researchers have carried out several studies and surveys on different mobile applications available for curtailing the spread of COVID-19. Privacy issues on contact tracing applications, level of user acceptability and level of information provided among others were the features investigated. A solution that helps researchers improve the existing applications was suggested. After 4 months (March to April, 2020) of discovery of COVID-19, the authors in [22, 23] reviewed the commercially available mobile application in mobile stores and those published in literatures. Search terminologies like COVID and COVID-19 to query the applications were used. A total of 103 applications in both Apple iTunes and Google Play stores were observed. Most common functions among these applications include contact tracing, self-test and news on COVID-19. Different types of applications focused on providing information and tracking health status. Their result exposed the need for applications that will take care of psychological health of the public by providing user-oriented features. The authors in [24] carried out a research aimed at helping healthcare institutions choose most befitting mobile application among the available ones for COVID-19 self-monitoring and awareness. The research is believed to help developers of mobile application to develop or modify their applications to get the best result as regards to COVID-19 applications. The authors in [24] also investigated the mobile health applications (mHealth) available in the play store for *Android* and IOS-based application store. The research was conducted between April and May, 2020, in USA using VPN to access applications with country restrictions. The applications were accessed and compared based on the following criteria: the knowledge it provides on COVID-19; tracing of COVID-19 cases; home surveillance and ability to consult with health authorities. A total of 223 COVID-19 mobile applications were studied. Some of the IOS-based applications included infographic classification of COVID-19 cases while some of the *Android* based have features such as remote monitoring. The applications were criticized for not providing enough educational resources on COVID-19. Users when choosing a mobile application are expected to choose ones that mostly meet their requirement. Unfortunately most applications surveyed lack user satisfaction. Between April to September 2020, health applications available for users in application stores were reviewed [25]. The aim was to study the application and classify them based on the purposes they were built for. The research was conducted using PRISMA-ScR guideline. Only applications dedicated for COVID-19 were listed in the categories of health and medical. The major features and general purpose were analysed; the summary was presented using descriptive statistics. A total of 298 health-related applications were downloaded, while 115 met the criteria for inclusion. A total of 77 applications were developed by government for the purpose of tracking user’s personal health. Others include creating awareness, monitoring health, managing exposures to the virus and for research purposes. The researchers believed that building an application that helps the citizens to track their health and access information is the key to curtailing the spread of the pandemic. They recommended that researchers should improve on the applications and governments to create awareness on using these applications. Many have also argued on the privacy concern of these applications especially the contact tracing applications. The authors in [26] analysed contact tracing applications to investigate their privacy features, design and consequences as well as underlying technologies (Bluetooth and GPS). After studying 13 applications from 10 countries, the researchers noted that contact tracing application for COVID-19 are still facing challenges of public acceptance. This is due to some technical hitches and privacy issues reported by the users of the applications. It was claimed by authors in [27] to have solved the issue of privacy concern among contact tracing application through block chain technology which provides the user with a complete control over their personal information as long as the data remains in the application. According to them, user’s data are encrypted, immutable and time stamped which make unauthorized access difficult and promote transparency. After conducting user experience survey on user experience and view of COVID-19-related applications the authors in [28] recommended the following improvements for COVID-19 application developers: a consolidated application to reduce the number of applications a user has to download and use; develop an easy to use application for users with less experience in using mobile application; inform users with useful information to avoid panic; protect user information and data; and adapt to the location and situation of the region at which the applications are utilized. Some of these recommendations have been implemented in CoFighter. Our research consolidated several functions like case report, case updates, global update check, vaccine status check, preventive measure information, etc., in one application. It is user oriented with its numerous user actions. In adapting the application to user’s location, the researchers made sure that users can check COVID-19 status of their cities.

### The research gap found

The research gaps found and the corresponding solutions provided in CoFighter are as follows:

- *Most applications have limited features* This implies that Users may need to install more than one application to get effective service. CoFighter integrated different features in one application. Such features include suspected death report, suspected case report, vaccine booking, vaccine status check, vaccine complication report, global case report, preventive measures, state or province case report and test result delivery email system.
- *Absence of vaccine information on existing applications* This research gap was solved by providing a mechanism that allow individual to book for vaccination and be assigned a vaccine ID. Users can report vaccination complication or side effects without visiting the hospital or vaccine centre through Vaccine Complication Report feature. A feedback is sent through user’s email address. Vaccination Status Check is used to check vaccination status of an individual by providing a booking ID. This eliminates vaccine certificates carried about by travellers as evidence of having been vaccinated.
- *There is no central system for COVID-19 workers to manage the cases* To get automatic update and increase the efficiency of case management, a web-based application was developed. This provides health workers with a platform to manage reported cases, complications, vaccination, discharge, deaths and confirmed cases. This web-based application communicates with the mobile application effortlessly.

## Analysis of the proposed system

The proposed system is an *Android* application for portability, efficiency and easy management of the COVID-19 and related pandemics. Our application can be used by an individual, frontline health workers and the government. It focused on providing user with important information on COVID-19 to enable them remain safe, access to vaccine information and live case updates. It provides a management system for front line workers that simplify their work. The proposed system uses some selected data on COVID-19 case management, vaccine centres and registration to demonstrate these features. The global case update comes from John Hopkins University public API on COVID-19 global case updates. This is achieved using a mobile-based *Android* application which is faster and easier to access when compared to web-based application. Users can help the government by reporting vaccine side effects, complications and suspected cases using CoFighter.

### Methodology

**Fig. 1:**
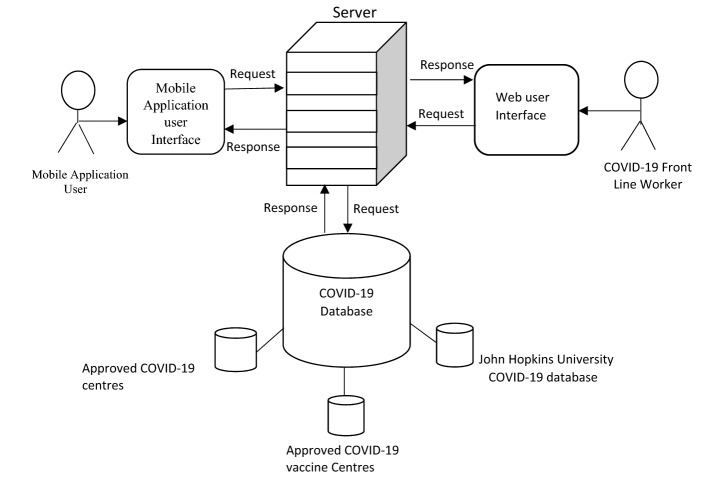
CoFighter system flow diagram

CoFighter has two different frontends, a backend and a database. The Frontend-Mobile application was built with Flutter technology, while the Frontend-Web application was developed with HTML, CSS and JavaScript. The Backend was developed with PHP, whereas the database used is MYSQL. We understand the role of a responsive and an attractive user interface (UI) in acceptability of a mobile application from users and thus, our choice of Flutter in defining or structuring the UI. The mobile application is available for easy download and use to access the various resources. This client side of the application is used by COVID-19 Front Line Workers and COVID-19 Vaccination workers to manage their day to day activities and update cases automatically. Figure 1 illustrates the system flow of CoFighter. Mobile Application users and COVID-19 frontline workers use mobile application interface and web interface, respectively, to access the server which accesses the COVID-19 database linked to John Hopkins University COVID-19 database and other approved COVID-19 centres and vaccination databases as shown in Fig. 1.

### Description of the system

The homepage of the proposed system displays the COVID-19 status of the country on the dash board of the mobile application. The status includes total number of confirmed cases, actives cases, discharged cases and death cases. From the homepage, users can quickly access the various COVID-19 prevention measures and view case updates without registering or logging into the application. The menu icon contains links to the following menus: Report COVID-19 Case, Get Vaccinated, Report Vaccine Complication, Check Vaccine Status and View Global Cases. The proposed system helps users to achieve the following:

1. Locate features and access it;
2. Allows user to report COVID-19 suspected case;
3. Provides a platform for checking vaccination and status of an individual;
4. Allows users to register for vaccination and be booked for it; and provides real-time update on COVID-19 cases.

### Features of CoFighter

The features of the application are as follows:

#### Report COVID-19 case

This feature enables both individual and third party to report suspected COVID-19 cases and suspected death to the COVID-19 frontline worker in COVID-19 centres. A reported case is registered and managed in the centres. Hospitals, other institutions and individuals can report a case for investigation. The frontline workers in the field get the report and use the address and contacts provided to trace the person if necessary or give advice. This prevents potential spread.

#### Check vaccine status

Individual, government, religion houses, games houses, social clubs, event centres and cooperate organizations can use this feature to confirm individual’s vaccination status accordingly. This replaces the idea of caring around vaccine certificate as a means of confirmation.

#### Register and book for vaccination

Since the development of the vaccine for COVID-19, the level of the acceptance from the developing countries, e.g. Africa has been underwhelming. There are lots of fake news circulating as regards to the vaccine. This feature provides easy and fast access to vaccine. Users can register or book for vaccine by selecting their preferred date and vaccine centre from their preferred cities.

#### Vaccine complication report

This feature helps individuals report side effect after taking the vaccine and get help as soon as possible. It helps health workers monitor and study the trend in the aftermath of vaccination and tackle it appropriately by improving the vaccine. User can choose among the selected potential side effects and also specify any other side effect experienced.

#### Preventive measures/news

This includes day-to-day COVID-19 measures, educative piece on COVID-19 pandemic and general health advice, coming from the COVID-19 centres.

### Operation flow of the application

The operation flow of the four different actors for mobile and web users is shown in sections "For mobile application users" and "For web application users".

#### For mobile application users

- The application is launched by clicking on the icon.
- The dashboard displays showing total number of cases
- Users can click a downward link to view a specific state or city cases
- Users can click the three parallel lines at the top right corner to view and select any of the menus or
- Choose the icons of the different menus down the screen for quick launch.
- Users exit the application by simply closing it.

#### For web application users

- The actors (super admin, admin and COVID-19 health worker) are expected to register according to their privileges.
- User launches the application by visiting the URL
- Login page appears asking for username and password.
- User enters credential
- The credential is compared and if valid grants access to the corresponding resource(s).
- If the login details are not found, user is asked to try again or register as a new user.

Actors exit the application after using it by logging out.

## Comparative analysis of CoFighter with other related applications

A comparison of CoFighter with other related applications is shown in Tables 1 and 2. While Table 1 compares CoFighter with other related application in terms of similarities and differences, Table 2 carries out a general comparison in terms additional functionalities of CoFighter.

**Table 1:** CoFighter compared with other applications in terms of similarity and differences

Author/year	Title	Platform	Method	Result	Features similar with CoFighter	Added features of CoFighter
Wissel et al. [12]	COVID-19 Watcher	Web application	R. Shiny Ggplot2	Provides Cases Update for USA. Returns info on testing, capacity, cases and deaths Ranks and plots the result	Provides cases update	Mobile application Provides case updates for other countries Individual can also report cases too
Saxena et al. [13]	COWAR	Mobile application	Java, Firebase	Live cases update (India). Check symptoms. Makes provision for people who wants to make donations	Live case update Mobile application	Method is Flutter, PHP, MySQL etc Report cases Manages Vaccination
Maghdid and Ghafoor [15]	Smartphone-Based Approach	Mobile application	PHP, JavaScript, HTML5	Contact tracing Makes lockdown prediction to guide government and travellers	PHP, Javascript, HTML5	Cases update Manages vaccination, Reports vaccine complications, Checks vaccination status
Hale et al. [16]	The Oxford COVID-19 Government Response Tracker (OxCGRT)	Web application	Azure SQL Database Stata	Tracking government policy during COVID-19 and its impact on containing the spread	Research is aimed at controlling the spread of COVID-19	Reports Live cases, Manages vaccination, Reports complication from vaccine
Sahu et al. [17]	Indian COVID-19 Tracker	Web/Mobile	Flutter JAVAScript	Public awareness Cases update Pictorial representation of cases	Cases update for India Similar method or technology	Manages vaccination, Reports cases, has global cases update

**Table 2:** Comparative analysis of CoFighter with other related applications

S/n	Application Name	Application developer	Technology (Android & iOS)	Real-time information	Case update feature	Scope of the application	Contact tracing feature	Availability of world data	Availability of vaccine information	Provision for test result Check
1	COWAR	Saxena et al	Android based	Information delivery is in real time	Has Live cases update	Available to the public	No contact tracing	World data not available	No vaccine information provided	No provision for test result
2	COVID-19 Tracker	Sahu et al	Android based	Information delivery is real time	Case update is provided	Available to the public	No contact tracing	Word data not available	Vaccine information not provided	Test result not included
3	TraceTogether	Government of Sinagpore	Android based and uses Bluetooth tech	Information delivery is in real time	No cases update provided	Used only in Singapore	Provides for contact tracing	Word data not available	Vaccine information is provided	Test result included
4	CovidWatch	University of Arizona	Android & IOS based	Information delivery is in real time	No case updates provided	Available only to US citizens	No contact tracing	Word data not available	Vaccine information not provided	Test result not included
5	AarogyaSetu	Government of India	Android & IOS based	Information delivery is in real time	Cases update is provided	Available to the public	Provides for contact tracing	Word data not available	Vaccine information not provided	Test result provided
6	Immuni application	Government of Italy	Android & IOS based	Information delivery is in real time	No cases update provided	Available to the public	Provides for contact tracing	Word data not available	Vaccine information not provided	Test result not included
7	COVIDSafe	Government of Australia	Android & IOS based; uses Bluetooth tech	Information delivery is in real time	Cases update is provided	Available only to Australian citizens	Provides for contact tracing	Word data not available	Vaccine information not provided	Test result included
8	CoFighter		Android & IOS based	Information delivery is in real time	Cases update is provided	Available to the public	No contact tracing	Word data is available	Vaccine information fully provided	Test result included via email message

## Results

Tables 1 and 2 show a comparative analysis of CoFighter with related applications. We conducted a comparative study among some popular mobile applications developed to fight COVID-19 by comparing them with CoFighter. We selected eight major important features that these applications needed to have for effective management and prevention of COVID-19. Some of the applications were downloaded to be able to access the features while some that have strong geographical restrictions were analysed based on their listed features from the literatures surveyed. Eight mobile applications were analysed and compared using eight different features as shown in Table 2. Ranking them, CoFighter came first with seven features out of the eight considered, AarogyaSetucame second with six features, TraceTogether and COVIDSafe tied in third position, Immuni application came fourth, COWAR and COVID-19Tracker also tied in fifth position, while COVIDWatch is in sixth position. Most of the mobile applications focused on contact tracing without doing much on the management of the cases and providing important information to the users.

CoFighter being a consolidated mobile and web-based application also provides for user case report, global case updates and use, vaccine information including booking for vaccination, reporting side effects and complications arising from vaccine to provision of vaccine certificate. Cofighter is available to individuals, frontline health workers and government for use. It provides rich prevention information to citizens and management information to government and health workers on covid-19 and related pandemic in a user friendly manner to avoid panic and crisis. COVID-19 test results are also made available to user via email.

### Screenshots from CoFighter

**Fig. 2:**
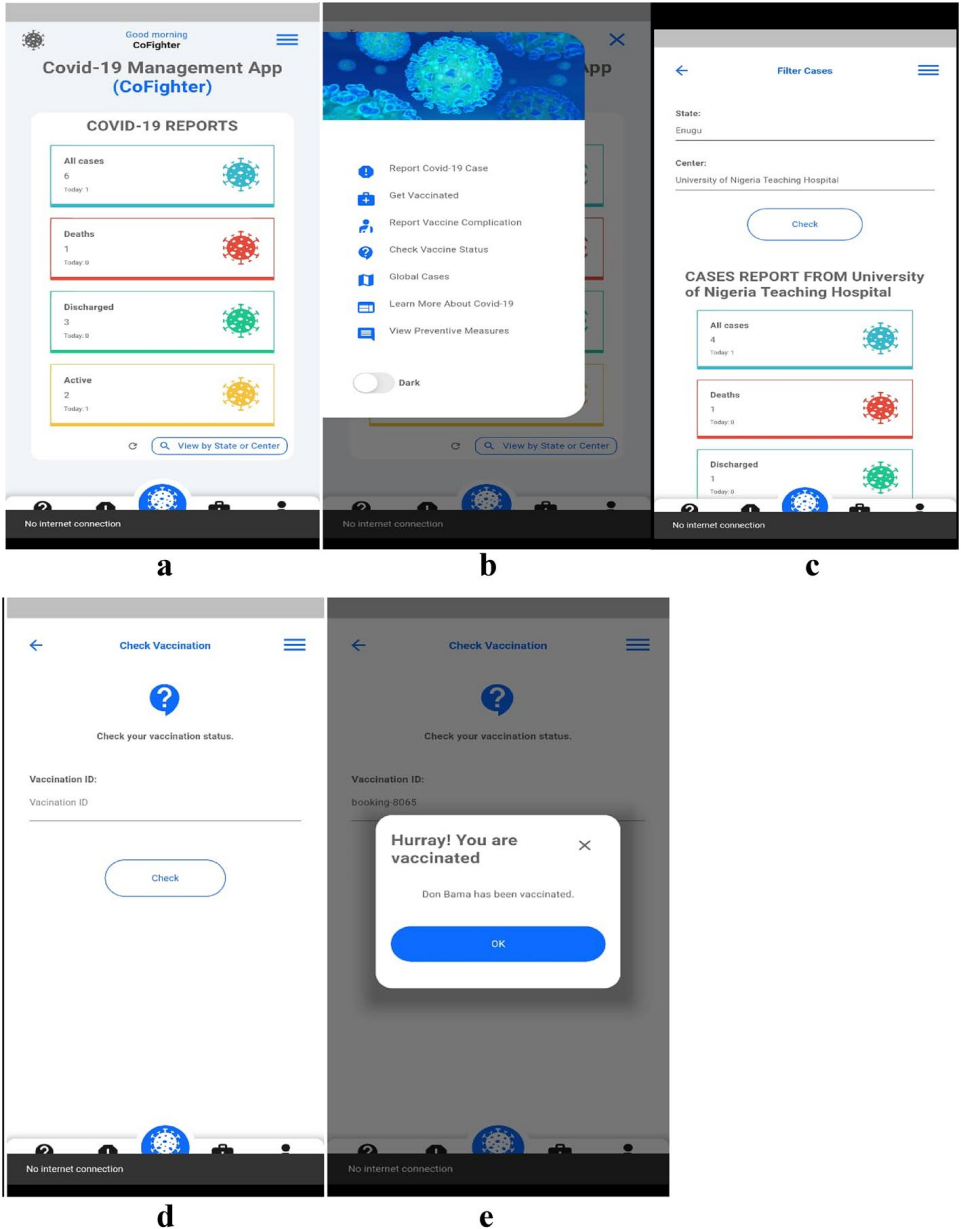

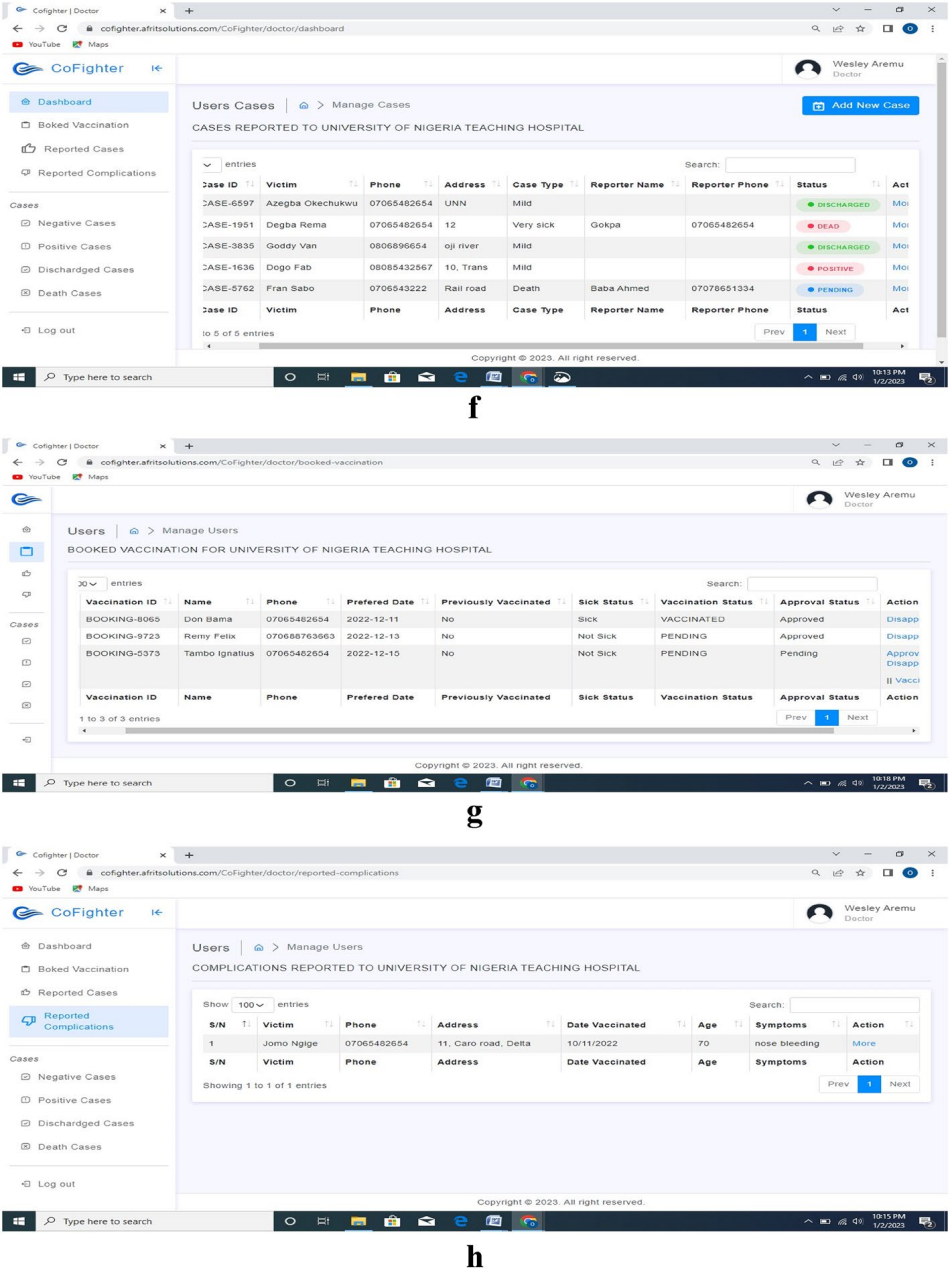
**a** CoFighter dashboard. **b** CoFighter Main menu. **c** Location search menu. **d** Check vaccination status. **e** Vaccination status report. **f** Frontline health workers dashboard. **g** Manage vaccination dashboard. **h** Vaccine complications dashboard

Figure 2a shows the dashboard of CoFighter with cases updates in real time. Users can access the quick menu button on the application or click on the three horizontal line at top right corner. Figure 2b shows different features available to the users of the mobile application. Figure 2c depicts result for state-wise search. Figure 2d is a platform for checking vaccination status of any individual by inputting the vaccine ID obtained during vaccine booking. Figure 2e shows vaccination status after successful vaccination. Figure 2f is a web-based application dashboard for COVID-19 health workers. The reported cases from the application are viewed and managed here. Authorized health worker can confirm positive or negative cases after test, discharged cases and confirmed deaths. These are updated on the mobile application accordingly and timely. Test results are sent to email address provided by patients as soon as they are confirmed. Figure 2g is the interface used to manage vaccination. Booked vaccination is reported on this dashboard and managed. Approved dates and vaccination centres is sent to the user through email address upon approval. Figure 2h shows the dashboard for reported vaccine complications or side effects. The frontline health workers use it to easily offer medical advice or prescriptions based on the symptoms reported after the vaccination. Cofighter therefore not only helps reduced panics due to side effects from vaccines, but also saves time and cost associated with revisiting the hospitals or centres.

## Conclusion

It does not look as if COVID-19 will be totally wiped out any soon at the moment as it is still ravaging China. The aim of CoFighter is to manage the pandemic effectively and at the same time, tame the spread. CoFighter consists of two platforms, the mobile application and the web application. The mobile application have the features that enable the populace report cases, book for vaccination, view live case updates, get breaking news, check vaccination status, report complications or side effect after vaccination and view other countries’ COVID-19 cases. The web application is a total management platform for COVID-19 frontline health workers for live update and management of cases, quick information on cases and vaccination management. The mobile application was developed using Flutter, while the web application was built with HTML5, CSS and JavaScript. The server side was built with PHP and MySQL as the database. The two platforms communicate with the hosted server through a Laravel API. The mobile application features are fast, simple with beautiful interface that easily get the user going while the web application contains self-explanatory features that may not require any explanation to the workers. This paper has also solved the issue of carrying paper certificate around as a means of vaccination identification through its check vaccine status feature.

## Recommendations

We recommend that the web application be stored in every COVID-19 centre for effective management of pandemics. The check vaccine status should be used at airports and other places where only vaccinated people are allowed to have access. The mobile application should be publicized by the government for wide acceptance.

### Suggested areas for further work

1. Developing a system that can effectively manage any outbreak.
2. Telemedicine should be incorporated for remote treatment of highly contagious disease.
3. In time of pandemic, people may lose access to the internet or find it difficult to access data. Developing an offline version of this mobile application will solve such a problem and could result to massive adoption and use of the application.

## Data Availability

The developed application, CoFighter, is available at https://github.com/OkeyIsOkay/CoFighter-Project.

## Abbreviations

COVID-19: Coronavirus disease 2019
SARS-CoV-2: Severe acute respiratory syndrome coronavirus 2
OOADM: Object-Oriented Analysis and Design Methodology
XML: Extensible Markup Language
PHP: Hypertext Preprocessor
MYSQL: A combination of "My", the name of co-founder Michael Widenius' daughter My and "SQL" Structured Query Language
WHO: World Health Organization
IDE: Integrated development environment
GNSS: Global Navigation Satellite System
MAC: Media Access Control
HTML: HyperText Markup Language
API: Application Programming Interface
OxCGRT: Oxford COVID-19 Government Response Task
APP: Mobile application
5G: Fifth Generation
mHealth: Mobile Health
IOS: IPhone Operating System
PRISMA-ScR: Preferred Reporting Items for Systematic reviews and Meta-Analyses extension for Scoping Reviews
GPS: Global Positioning System
CSS: Cascading Style Sheets
UI: User Interface
URL: Uniform Resource Locators
